# Strong agreement in absolute marine prokaryote abundance from epifluorescence microscopy and internal-standard-corrected metagenomics

**DOI:** 10.1093/ismeco/ycag297

**Published:** 2026-10-14

**Authors:** Qicheng Bei, Daria Di Blasi, Nathan L R Williams, Abigail Mallo, Jelani Williams, Jed A Fuhrman

**Affiliations:** Department of Biological Sciences, University of Southern California, Los Angeles, CA 90089, United States; Department of Biological Sciences, University of Southern California, Los Angeles, CA 90089, United States; Department of Biological Sciences, University of Southern California, Los Angeles, CA 90089, United States; Department of Biological Sciences, University of Southern California, Los Angeles, CA 90089, United States; Department of Biological Sciences, University of Southern California, Los Angeles, CA 90089, United States; Department of Biological Sciences, University of Southern California, Los Angeles, CA 90089, United States

**Keywords:** quantitative metagenomics, epifluorescence microscopy, SYBR green, cell counts

## Abstract

Quantifying microbial cell abundance in natural environments with taxonomic resolution remains a central challenge in microbial ecology. While epifluorescence microscopy reliably enumerates free-living cells, it lacks taxonomic specificity and is less suitable for quantifying cells in sediments, soils, and on surfaces. Furthermore, most omics approaches profile microbial communities, but they typically provide only relative abundances. Recent advances in quantitative metagenomics using single-copy marker genes and genomic internal standards enable taxon-specific abundance estimates in absolute units (e.g. haploid genome equivalents L^−1^). Here, we directly compared SYBR Green–based epifluorescence cell counts with spike-in metagenomic estimates derived from bacterial *recA* and archaeal *radA* gene abundances in seawater samples collected monthly at five depths (5 to 890 m) from the San Pedro Ocean Time-series (SPOT) during 2023–2024. Across all depths, quantitative metagenomic estimates of prokaryotic abundance closely matched SYBR Green-based counts (slope = 1.05; Pearson’s *r* = 0.85). In addition to validating total abundance, quantitative metagenomics provided high-resolution, taxon-specific estimates across depths and sampling dates. The strong agreement between microscopy cell counts and metagenomic estimates from unfractionated SPOT samples suggests that most prokaryotes were free-living rather than particle associated. These results validate quantitative metagenomics as a robust approach for estimating absolute prokaryotic abundance, establishing a framework for taxonomically resolved quantification in microbial ecology and ecosystem modeling.

A longstanding challenge in microbial ecology is quantifying microbial cell abundance in natural environments with high taxonomic resolution. Direct counting methods, such as epifluorescence microscopy using DNA stains, enable visualization and enumeration of microbial cells, particularly planktonic populations [1]; however, DNA-staining approaches provide limited taxonomic resolution for most prokaryotes. High-throughput sequencing has greatly advanced the characterization of microbial community composition, especially in soils and structurally complex environments where optical methods are impractical, yet omics approaches typically yield only relative abundances. Targeted methods, such as fluorescence in situ hybridization and quantitative polymerase chain reaction (PCR), can quantify specific taxa but generally do not provide a comprehensive view of entire microbial communities [2], while relative proportions derived from 16S/18S rRNA gene surveys can be misleading [3]. In oceanography, absolute abundances are essential for quantifying microbially mediated biogeochemical processes [4] and for ecosystem modeling [5].

To overcome the limitations of relative abundance estimates, known quantities of nucleic acids, called internal standards (ISDs) or spike-ins, can be added to samples during DNA extraction. ISD recovery ratios enable the estimation of absolute gene abundances in amplicon sequencing [6]. However, rRNA gene copy numbers per genome vary widely and remain largely unknown for many taxa, potentially contributing to systematic offsets between amplicon-based abundances and microscopy cell counts [7]. Recent advances in quantitative metagenomics have circumvented this limitation by targeting single-copy marker genes and incorporating genomic ISDs, thereby enabling abundance estimates in absolute units (e.g. genome equivalents L^−1^) [8]. For example, Gifford et al. applied *recA*-based metagenomics to quantify prokaryotic populations near the Galápagos during an El Niño event [9]. Our previous spike-in metagenomics closely matched cyanobacterial flow cytometry counts (based on *recA*), although agreement was moderate for photosynthetic eukaryotes (based on *psbO*) [10], highlighting remaining challenges in quantifying diverse eukaryotic groups [11]. Despite these advances, independent validation of quantitative metagenomics remains essential to assess their accuracy and robustness. In particular, the relationship between spike-in metagenomic estimates and epifluorescence counts from surface to mesopelagic depths has not been systematically evaluated, although Gifford et al. reported general agreement for summed *recA* abundances in surface seawater samples [9].

Taking advantage of time-series sampling at SPOT, we quantified absolute prokaryotic abundances using single-copy marker genes (*radA* for archaea, *recA* for bacteria) and directly compared them with epifluorescence cell counts. Seawater samples were collected monthly during 2023–2024 from five depths: 5 m, deep chlorophyll maximum (DCM; typically 15–45 m), 150, 500, and 890 m (Table S1). Approximately 1 L of seawater was filtered onto 0.22 μm Sterivex filters for DNA extraction, and genomic ISDs (*Thermus thermophilus, Blautia producta*, and *Deinococcus radiodurans*) were added to crude extracts immediately after bead beating, as described previously (Table S2) [11]. In parallel, seawater samples (2–5 mL, depending on depth) were vacuum-filtered onto 0.02 μm Anodisc filters, stained with SYBR Green I, and enumerated by epifluorescence microscopy (in duplicate) [12]. SYBR Green stains both viruses and cellular particles; prokaryotic cells were distinguished from viral particles based on their larger size and defined cellular morphology (Fig. 1A). In practice, nearly all cells observed in randomly selected fields were either isolated or present in small clusters, rather than attached to larger particles. Absolute abundances derived from quantitative metagenomics (haploid genome equivalents L^−1^) were directly compared to microscopy-based prokaryotic cell counts (cells L^−1^). See Supplementary Methods and Figs S1–S3 for additional details.

**Figure 1:**
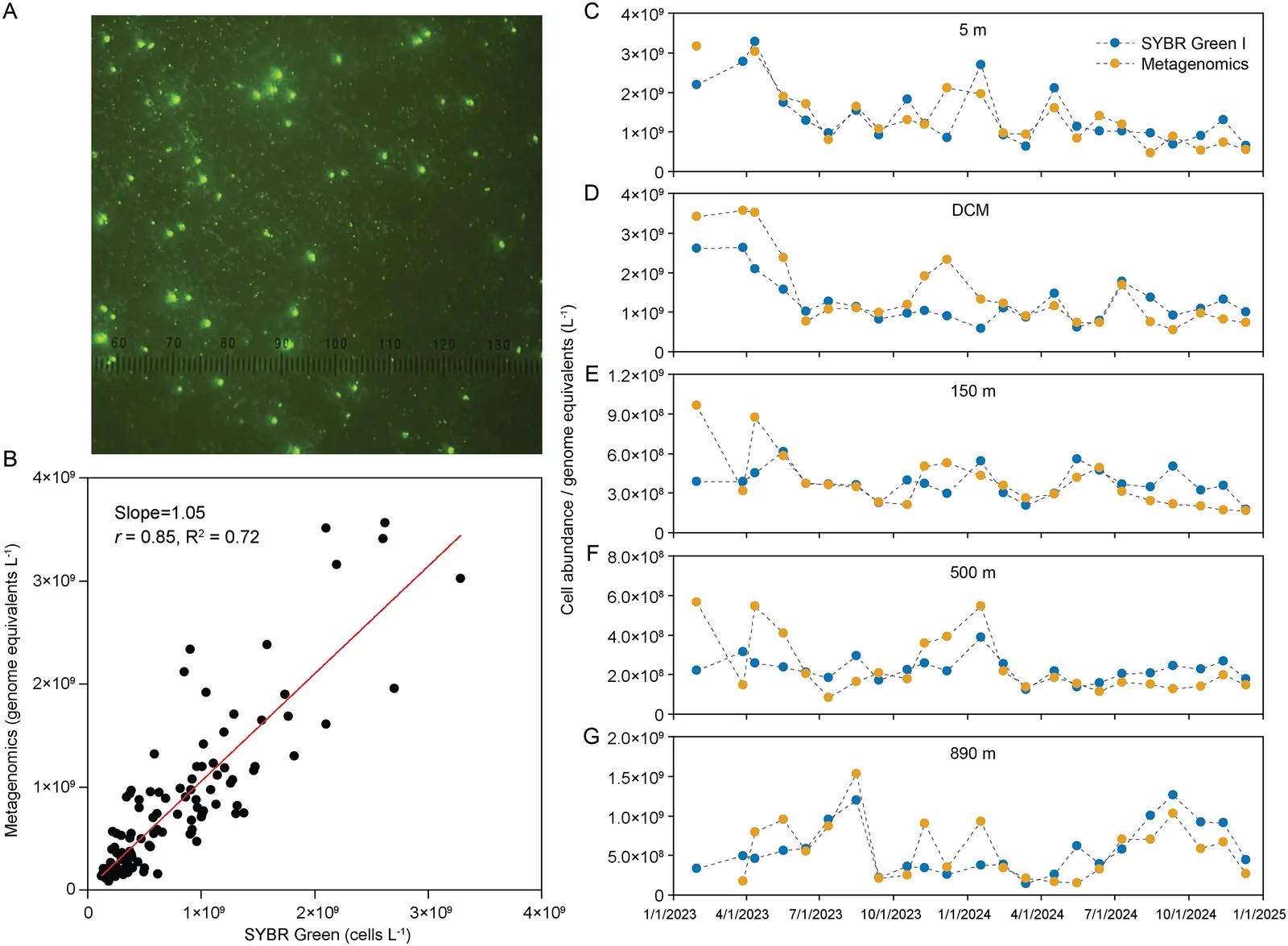
Validation of quantitative metagenomics against epifluorescence microscopy. (A) Representative SYBR Green–stained micrograph of a seawater sample collected at 890 m depth (scale bar unit = 1.0 μm). SYBR Green I stains double-stranded DNA, allowing visualization of both viral particles and microbial cells; prokaryotic cells were distinguished based on size and morphology. (B) Comparison of quantitative metagenomic estimates of prokaryotes (haploid genome equivalents L^-1^) and SYBR Green–based cell counts (cells L^-1^) across all sampling depths and dates. (C–G) Comparisons of SYBR Green–based counts and metagenomic estimates at 5 m (C), the deep chlorophyll maximum (DCM; D), 150 m (E), 500 m (F), and 890 m (G) depths at SPOT during 2023–2024. SYBR counts represent the mean of two replicate filters per sample.

Quantitative metagenomic estimates of total prokaryotic abundance, derived using genomic ISDs and single-copy marker genes, showed strong linear agreement with epifluorescence counts across depths and sampling dates (slope = 1.05; Pearson’s *r* = 0.85, *P* < 0.001) (Fig. 1B). Correspondence was strongest in surface (*r* = 0.76) and DCM (*r* = 0.77) samples, with moderate deviations at midwater depths (150 and 500 m) (Fig. 1C–G, Fig. S4), potentially reflecting incomplete marker-gene database coverage for poorly studied taxa in the mesopelagic zone [13]. Absolute abundances declined from a surface average of 1.36 × 10^9^ to 2.39 × 10^8^ cells L^−1^ at 500 m, while counts at 890 m (~10 m above the seafloor) averaged 5.90 × 10^8^ cells L^−1^, consistent with long-term SPOT observations [14]. The near-unity slope suggests that metagenomic genome equivalents provide a robust proxy for community abundance, despite documented variation in prokaryotic genome copy number associated with ploidy and growth stage [15]. The convergence of cell-based and sequencing-based quantification approaches supports the reliability of absolute abundances derived from two fundamentally different methodologies.

SYBR Green has long been used to enumerate microbial cells and dsDNA viruses in aquatic systems, and the strong agreement observed here with metagenomics suggests that prokaryotic cells were reliably distinguished from viral particles. While previous validations of quantitative metagenomics focused on *Prochlorococcus* and *Synechococcus* [16], our results demonstrate robust agreement across bacterial and archaeal communities, including the archaea-rich mesopelagic zone, where archaea can account for more than 25% of prokaryotic cells (Fig. 2). This agreement also provides a practical basis for optimizing spike-in strategies, including genomic ISD selection, standardization of spike-in timing, and refinement of DNA extraction protocols, thereby enhancing quantitative reliability across diverse environments.

**Figure 2:**
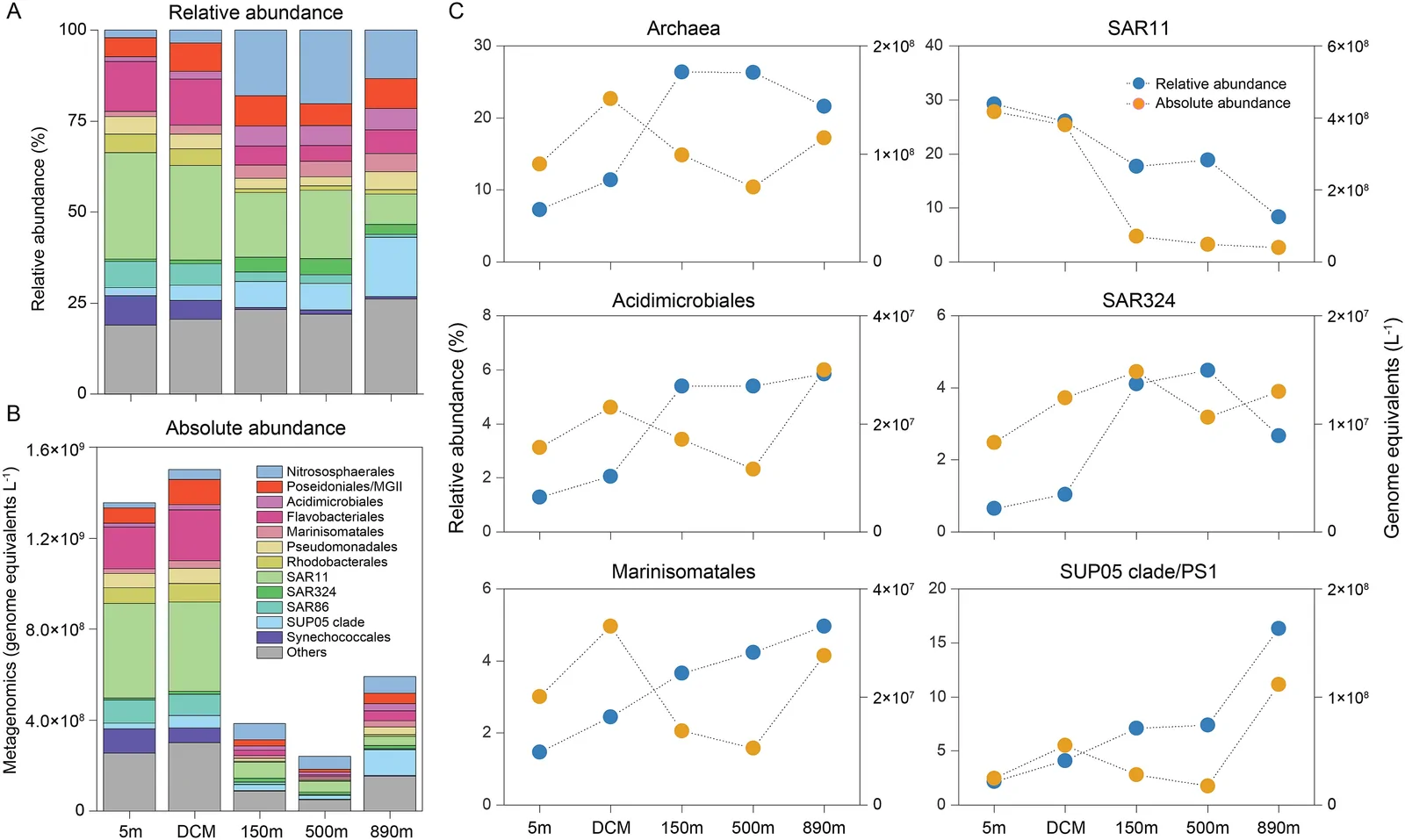
Taxon-resolved relative and absolute abundance profiles derived from quantitative metagenomics. Two-year average relative (A) and absolute (B; haploid genome equivalents L^−1^) abundances of prokaryotes at the order level across depths at SPOT during 2023**–**2024. Only taxa with average relative abundance >2% are shown; remaining taxa are grouped as “others”. Absolute abundances were estimated using spike-in metagenomics based on single-copy marker genes (*recA* for bacteria and *radA* for archaea) and genomic internal standards. (C) Comparison of relative and absolute abundance patterns for representative prokaryotic taxa across depths. Monthly absolute abundance profiles corresponding to panel B are shown in Fig. S5, while Fig. S6 demonstrates ecotype-level resolution.

Beyond validating total abundance, quantitative metagenomics provided high taxonomic resolution and revealed depth-dependent patterns that often contrasted with relative abundance profiles. For example, archaeal relative abundances were highest at 150 and 500 m, whereas absolute abundances were highest at the DCM (Fig. 2A and B). Similarly, the SUP05 clade (PS1 in GTDB taxonomy) exhibited higher relative abundances at 150 and 500 m than at the DCM, whereas their absolute abundance was higher at the DCM (Fig. 2C). Quantitative metagenomics also enabled taxon-specific absolute abundance estimation (Fig. 2 and Fig. S5), including ecotype-level resolution for cyanobacteria (Fig. S6).

Collectively, our findings demonstrate that spike-in quantitative metagenomics yields reliable estimates of absolute prokaryotic abundance. We expect this approach to apply not only to readily counted free-living cells but also to particle-associated cells, whose DNA can be recovered through bead beating during extraction [17]. By aligning closely with “gold standard” microscopy, quantitative metagenomics integrates high-resolution taxonomic information with absolute cell counts. Such capability is particularly valuable for quantifying microbial contributions to biogeochemical fluxes and refining ecosystem models that require absolute abundance data.

## Supplementary Material

Supplementary_Materials_ycag297

## Data Availability

Raw metagenomic data are available in the NCBI under PRJNA1426745. Databases of the single-copy genes (*radA* and *recA*) used for this study can be accessed through Figshare (doi:10.6084/m9.figshare.31302082).
