# Supplementary material for: Strong agreement in absolute marine prokaryote abundance from epifluorescence microscopy and internal-standard-corrected metagenomics
Source: ISME Commun. 2026 Oct 14;6(1):ycag297. doi: 10.1093/ismeco/ycag297 (PMC13747008; doi:10.1093/ismeco/ycag297)
Supplement: Supplementary file 1 [file supplementary_materials_ycag297.zip › Supplementary_Materials_ycag297/ISMEcommu-FigureS1-S6_ycag297.pdf]

Supplementary Information for

Strong agreement in absolute marine prokaryote abundance from epifluorescence microscopy and internal-standard-corrected metagenomics

Qicheng Bei*, Daria Di Blasi, Nathan LR Williams, Abigail Mallo, Jelani Williams, Jed A Fuhrman*

Department of Biological Sciences, University of Southern California, Los Angeles, CA90089, California, United States

*Corresponding authors: Department of Biological Sciences, University of Southern California, 3616 Trousdale Parkway, Los Angeles, CA 90089, California, United States.

Email: qbei@usc.edu; fuhrman@usc.edu

This PDF file includes:

Supplementary Methods

Figures S1 to S6

Supplementary Methods

1. Sample collection and epifluorescence microscopy

Sample fixation and filtration

Seawater samples were collected monthly from five depths (5 m, deep chlorophyll maximum [DCM], 150 m, 500 m, and 890 m) at the San Pedro Ocean Time-series (SPOT) during 2023–2024. Upon arrival at the laboratory, 49 mL of seawater was immediately fixed with 1 mL of 0.2-µm-filtered 2% formalin [1, 2]. Fixed samples were kept on ice until filtration the same day.

Duplicate filters were prepared for each sample. Two mL of seawater were filtered for the 5 m and DCM samples, whereas 5 mL were filtered for samples collected at 150, 500, and 890 m. Filtration was performed under vacuum using a glass filtration unit. A 0.8 µm Millipore AA membrane filter was pre-wetted with deionized water and used as a backing filter, and a 0.02 µm, 25-mm Anodisc filter (Whatman, 6809-6002) was placed on top. Excess liquid was gently removed from the underside of Anodisc by blotting without disturbing the surface.

SYBR Green I staining and slide preparation

A SYBR Green I working solution was freshly prepared in a light-protected tube by diluting 3.5 µL stock SYBR Green I in 1396.5 µL deionized water. An antifade mounting solution was prepared using 50% glycerol, 50% PBS, and 0.1% p-phenylenediamine (PPD) by mixing 7.2 µL of 10% PPD into 720 µL of a 1:1 PBS:glycerol mixture.

Following filtration, Anodisc filters were dried on a heat block for 2 min. Each filter was then placed sample-side up on 100 µL of diluted SYBR Green I and stained for 15 min in the dark at

room temperature. After staining, excess solution was removed by blotting from the underside of the filter, and filters were dried for an additional 2 min. Filters were mounted on microscope slides with approximately 50 µL of antifade solution and covered with a coverslip.

Epifluorescence microscopy enumeration and counting

SYBR Green I–stained filters were examined using an Olympus BX60 epifluorescence microscope equipped with a 100× fluorescence oil-immersion objective, a 150 W xenon lamp (Opti-Quip), and a U-MWB wide-band blue excitation filter cube (excitation BP 450–480 nm; dichroic mirror, 500 nm; emission barrier filter, 515 nm) (Fig. S1).

Cell density (cells/mL) was calculated by extrapolating the average cell counts per field to the total effective filtration area (19 mm diameter, area = 2.84×10^8 μm^2) using a reticle scale factor (RSF = 33,554, representing the ratio of total filterable area to the 10 × 10 eye-piece grid area), divided by the filtered seawater volume. For each replicate filter, cells were counted in 10 randomly selected fields (30-100 boxes depending on seawater depth) with at least 30 cells. Cell abundance was calculated from the mean number of cells, the effective filtration area, and the volume of seawater filtered, and expressed as cells/mL = 33,554 × Average count × (100/Boxes)/Volume.

Counts from the two replicate filters were averaged for comparison with quantitative metagenomic estimates. Prepared slides were stored at -20°C until enumeration, and remaining fixed seawater was stored at -80°C. When enumeration of prokaryotic cells alone is required, acridine orange may provide a simpler and lower-cost alternative.

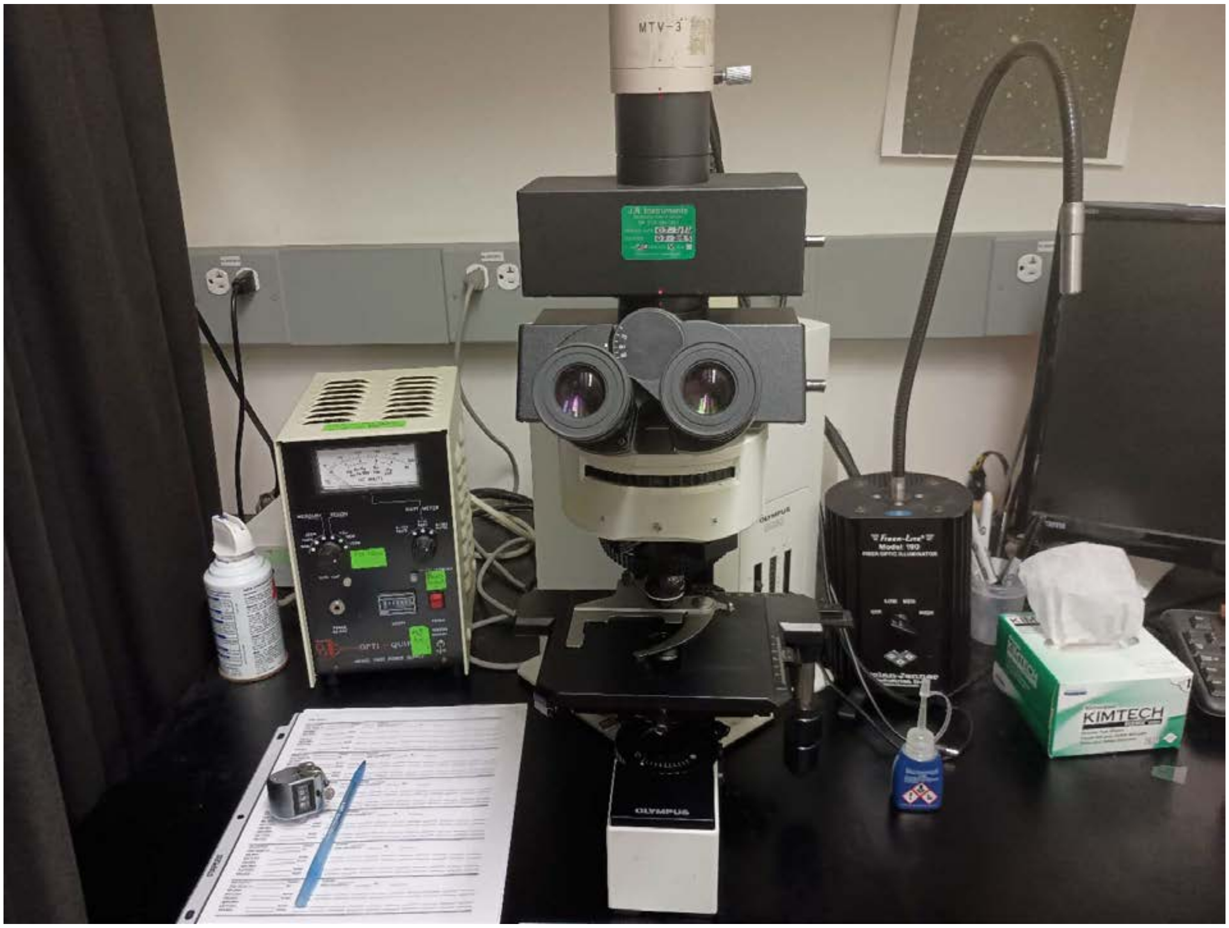

Figure S1. Epifluorescence microscopy setup. Epifluorescence microscopy workstation featuring an Olympus BX60 microscope equipped with an Opti-Quip Model 1600 xenon arc lamp power supply.

2. Quantitative metagenomics for marine prokaryotes

DNA extraction and internal standards

Total DNA was extracted using the AllPrep DNA/RNA mini kit (Qiagen, Valencia, CA, USA) with modifications for Sterivex samples. Briefly, the RNA/DNA shield (Zymo Research) was recovered from the Sterivex filter by brief centrifugation and recombined with the extraction lysis buffer. Sterivex filters were aseptically opened in petri dishes using flame-sterilized pliers. The filter membranes were removed from plastic housing, sliced into strips using sterile blades and forceps, and added to bead beating tubes containing 0.1-, 0.2-, and 0.5-mm zircon beads and RLT lysis buffer from the kit. Cells were lysed using bead beating. To determine total raw DNA yield (ng L^{-1}) prior to spike-ins, an aliquot of lysate was mixed with 8 µL of Polyacryl Carrier (PC152, Molecular Research Center) for DNA precipitation and quantified using a Qubit 4 Fluorometer with the Qubit dsDNA High Sensitivity Assay Kit.

For absolute quantitative analysis, three internal genomic standards (ISDs) (*Thermus thermophilus* ATCC BAA-163, *Blautia producta* ATCC27340, *Deinococcus radiodurans* ATCC13939) were spiked into the lysis buffer after bead beating, targeting ~1% of total DNA content [3]. Three ISDs were used to account for potential standard-specific variation in recovery. Because recovery of individual standards varied among samples/extractions, absolute abundances were calculated from the mean recovery ratio of the three ISDs rather than relying on a single genome throughout the study (Fig. S2). DNA purification was performed according to the manufacturer's protocol.

Metagenome sequencing and absolute quantification

DNA libraries were prepared and barcoded using the NEBNext® Ultra™ II FS DNA Library Prep Kit (New England Biolabs). The metagenomic libraries were sequenced an AVITI sequencer (2 × 300 bp mode) at the University of Minnesota Genomics Facility (UMGC). For metagenomic estimation of absolute cell abundances (haploid genome equivalents L^{-1}), we followed the previous study [3]. Briefly, raw reads were quality-trimmed using Trimmomatic v0.39 (ILLUMINACLIP:TruSeq3-PE.fa:2:30:10:8:TRUE LEADING:5 TRAILING:5 SLIDINGWINDOW:5:20 MINLEN:50) [4]. Paired-end reads were merged using PEAR v0.9.6 with default parameters (with minimum length 150bp) [5]. Reads from the internal genomic standards were identified via BLASTn (-evalue 1e-5, -perc_identity 95, -qcov_hsp_perc 50), followed by BLASTx searches (-evalue 1e-5).

Bacterial *recA* and archaeal *radA* proteins were downloaded from the NCBI protein database, cross-checked by GhostKOALA (https://www.kegg.jp/ghostkoala/) and manually curated (https://doi.org/10.6084/m9.figshare.31302082). Metagenome reads were compared to the databases using DIAMOND v2.1.9 (BLASTx, -e 1e-5, --id 60/70/80, --query-cover 60, --min-score 50) [6]. To evaluate the sensitivity of quantitative abundance estimates to marker-gene assignments, *recA* and *radA* homologs were identified using DIAMOND BLASTX at 60%, 70%, and 80% amino acid identity thresholds while holding the same query-coverage criteria. The relationship with SYBR-based cell counts remained stable across thresholds (Pearson's $r = 0.85$), while regression slopes ranged from 1.14 at 60% identity to 1.05 at 80% identity (Fig. S3a−3b). An 80% identity threshold was then used for the final analyses to increase marker gene assignment stringency. The taxonomies of *recA* and *radA* genes were determined by aligning the sequences to the NCBI nr database using DIAMOND (BLASTx, -e 1e-5, -k 25), and the outputs were summarized using MEGAN v7 community edition [7] with the GTDB taxonomy [8].

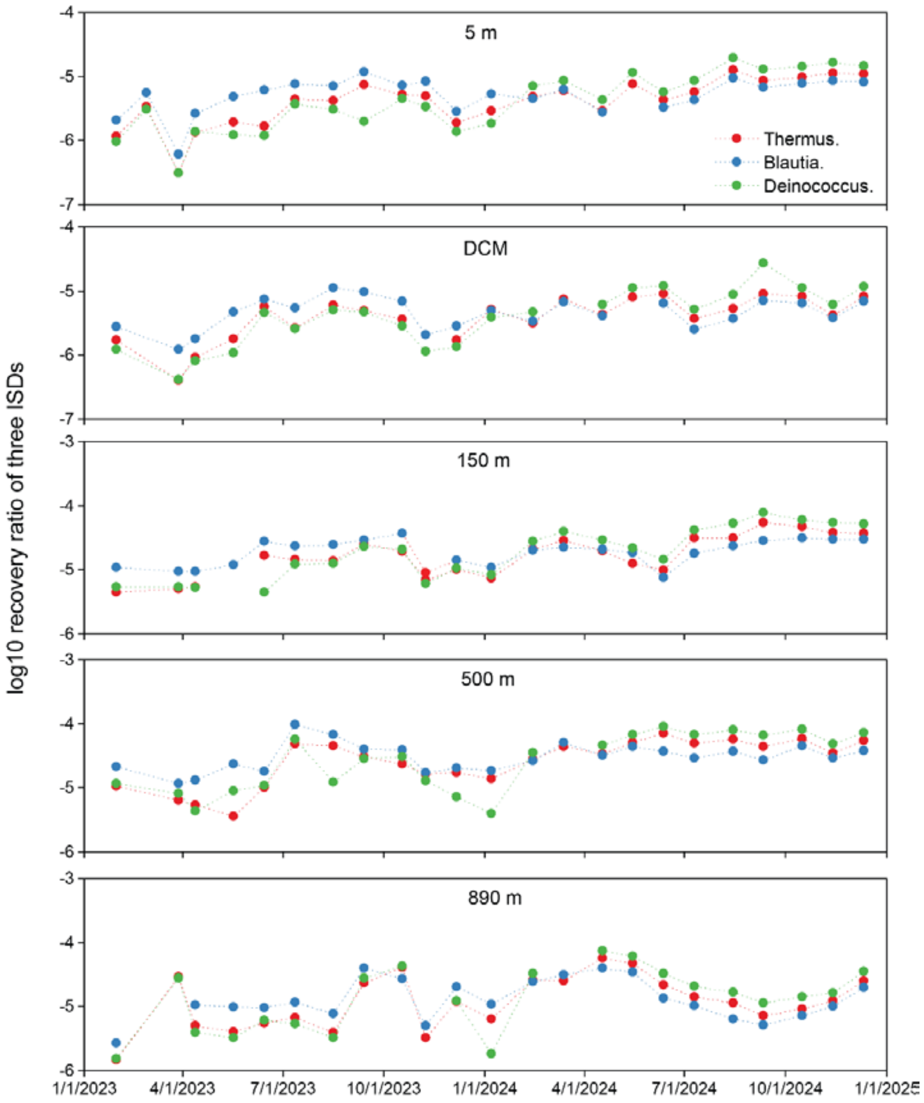

Figure S2. Log10-scaled recovery ratios of the three internal standards in SPOT samples at different depths. Recovery ratios varied among individual ISDs and processing batches (Table S2). The mean recovery ratio of three genomic ISDs was used for quantitative metagenomic abundance estimates throughout this study.

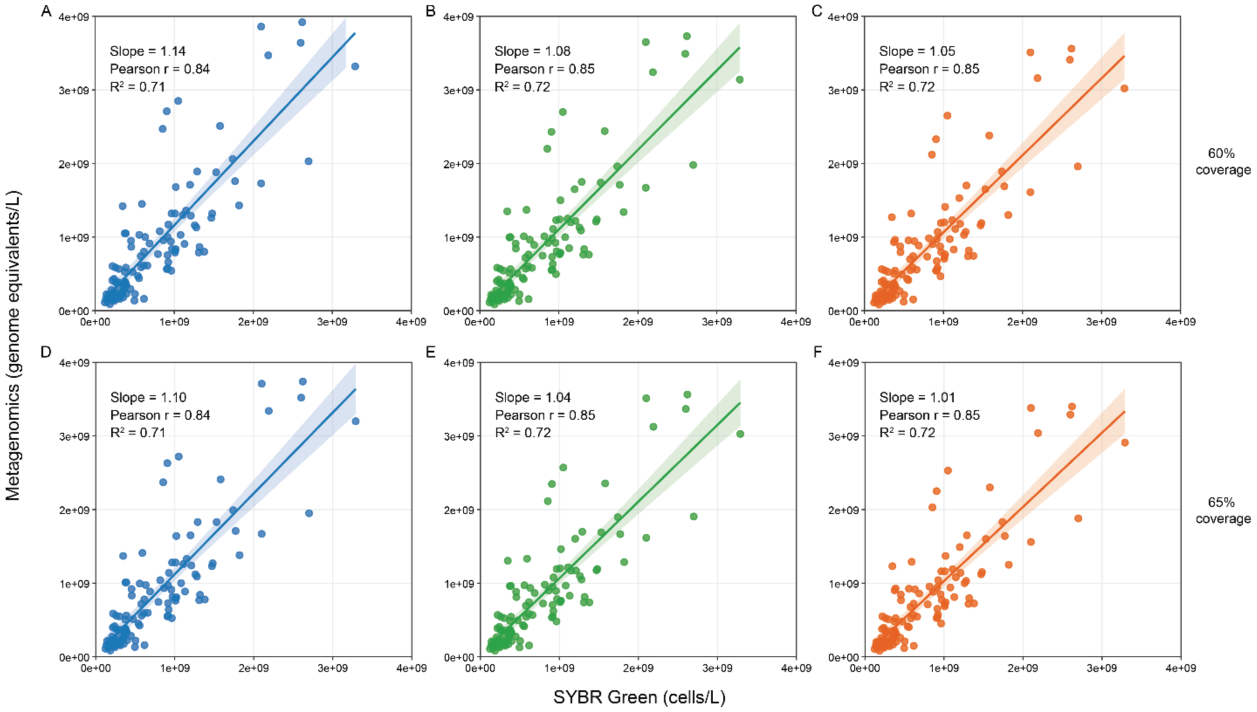

Figure S3. Comparison of DIAMOND blastx identity and coverage thresholds against SYBR Green cell counts across all samples. Correlation between SYBR Green-based cell counts (cells L^{-1}) and quantitative metagenomic estimates (haploid genome equivalents L^{-1}) calculated using three identity cutoffs in DIAMOND Blastx: (A–C) 60%, 70%, and 80% identity cutoffs with a 60% coverage threshold, and (D–F) 60%, 70%, and 80% identity cutoffs with a 65% coverage threshold for the single-copy marker genes *radA* and *recA*. Solid lines indicate linear regressions, with statistics provided in each respective panel.

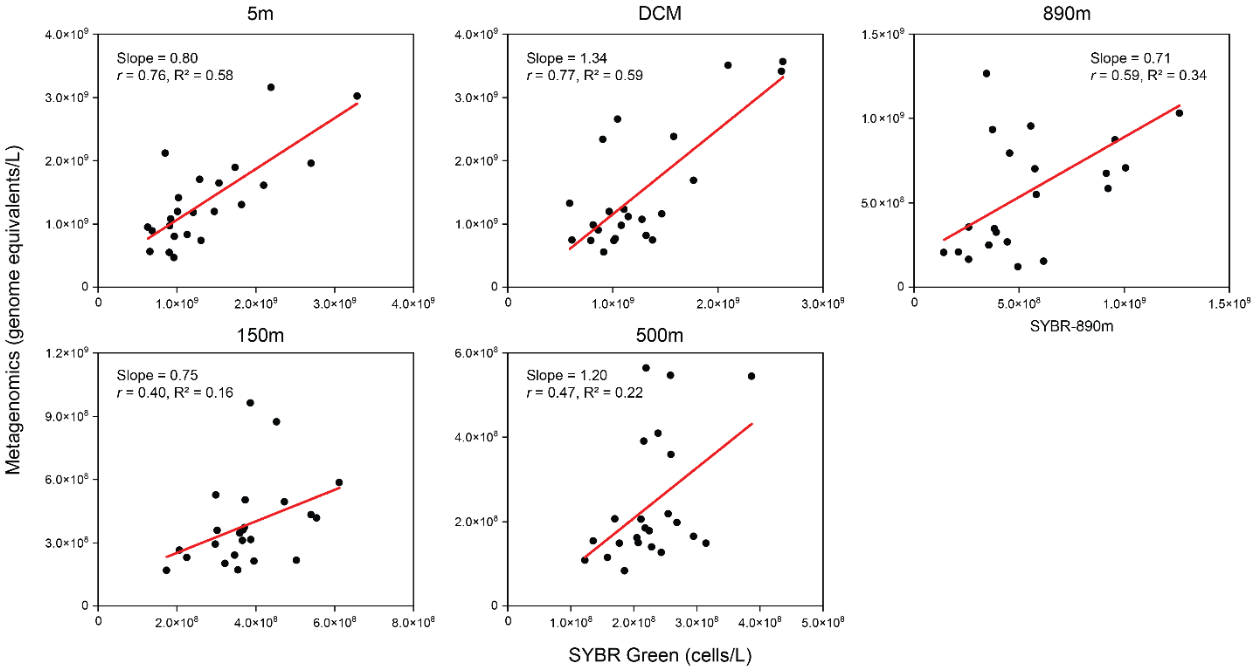

Figure S4. Depth-resolved comparison of quantitative metagenomic estimates and SYBR Green–based cell counts. Comparison of quantitative metagenomic estimates of prokaryotic abundance (haploid genome equivalents L^{-1}) and SYBR Green–based cell counts (cells L^{-1}) across depths and sampling dates. Seawater samples were collected at 5 m, DCM, 150 m, 500 m, and 890 m at SPOT during 2023–2024. SYBR counts represent the mean of two replicate filters per sample. Lines indicate linear regression fits for each depth (Diamond Blastx, with 80% identity and 60% coverage threshold). Correlation coefficients and slopes are shown in each panel.

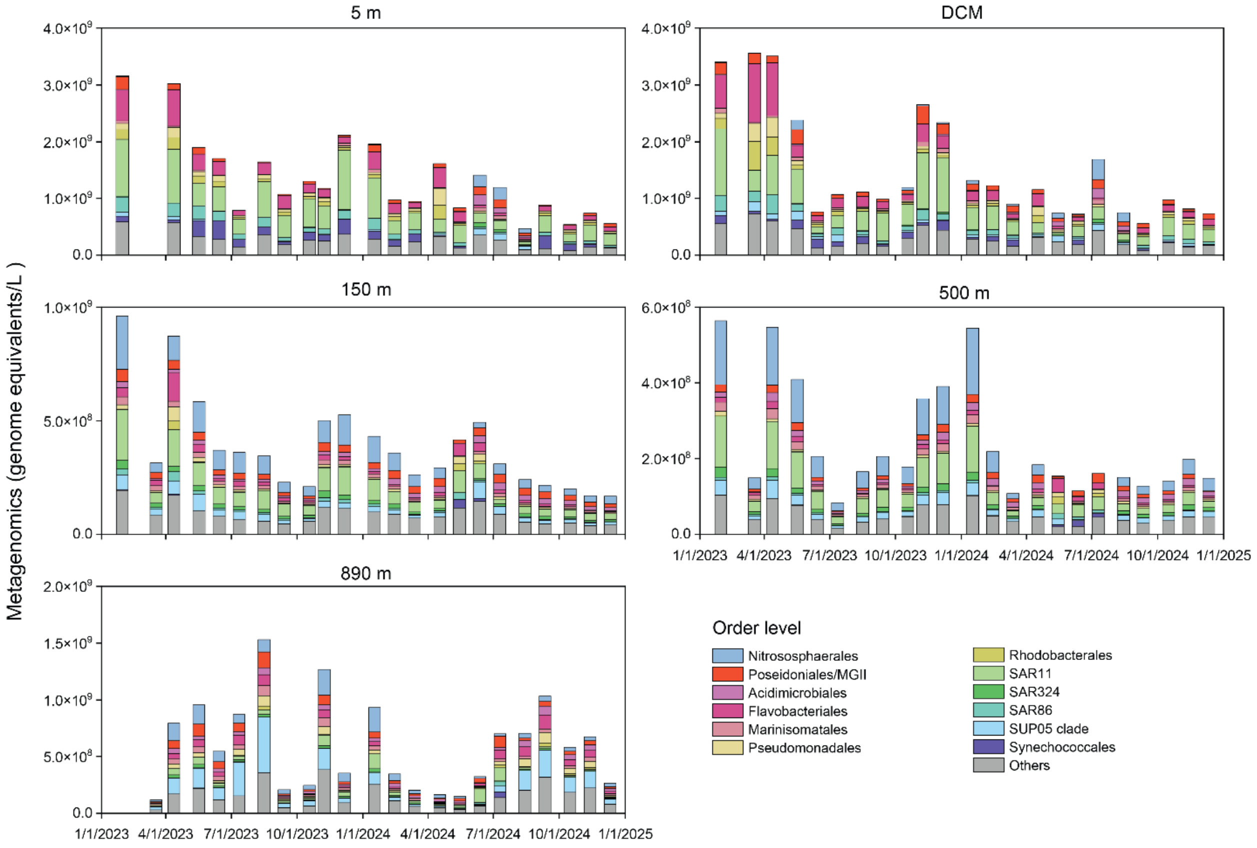

Figure S5. Taxon-resolved absolute abundance profiles derived from quantitative metagenomics. Depth-resolved time series of absolute abundances (haploid genome equivalents L^{-1}) of prokaryotic taxa at SPOT during 2023–2024. Panels correspond to 5 m, DCM, 150 m, 500 m, and 890 m depths. Only taxa with average relative abundance > 2% are shown; remaining taxa are grouped as “Others”. Absolute abundances were estimated using spike-in quantitative metagenomics based on single-copy marker genes (*recA* for Bacteria and *radA* for Archaea) and three genomic internal standards.

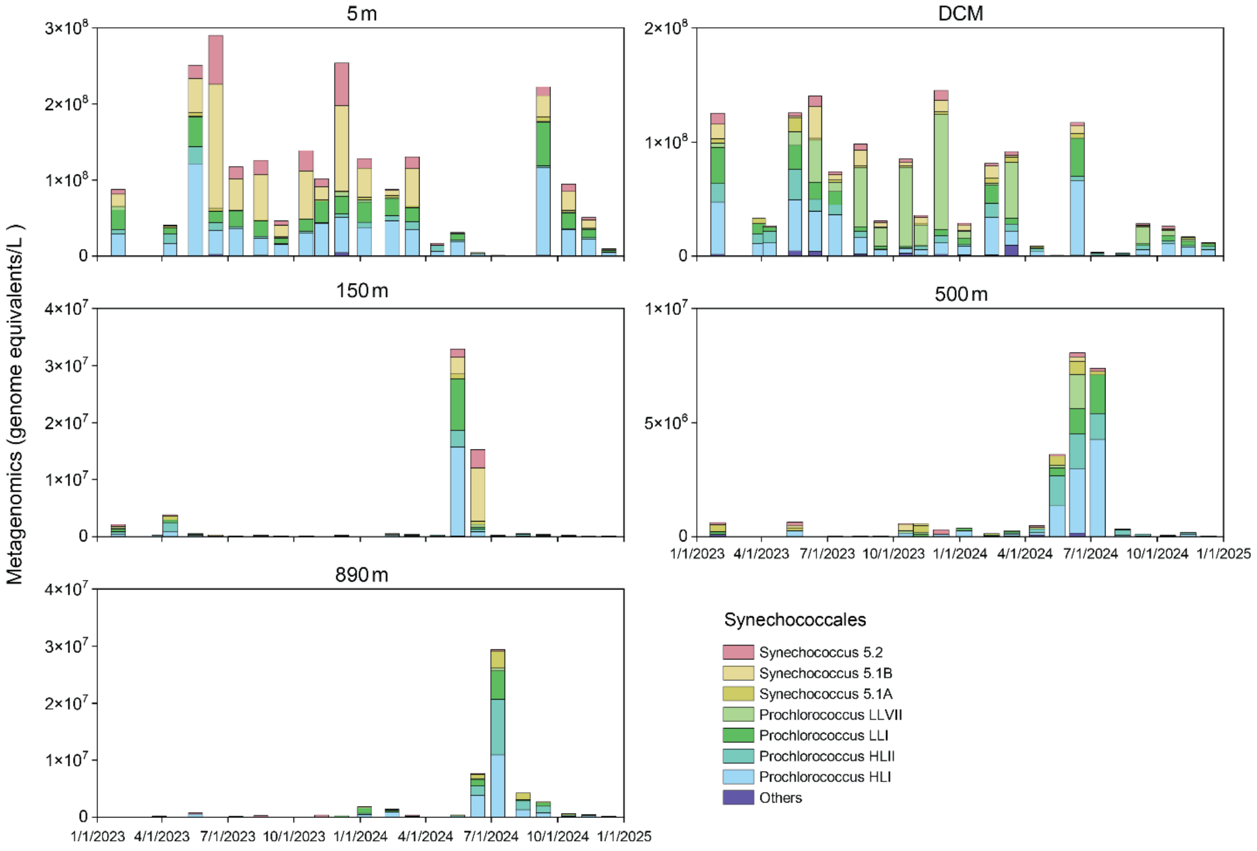

Figure S6. Absolute abundance of ecotype-resolved *Synechococcus* and *Prochlorococcus* derived from quantitative metagenomics. Depth-resolved time series of absolute abundances (haploid genome equivalents L^{-1}) of order *Synechococcales* at SPOT during 2023–2024. Panels correspond to 5 m, DCM, 150 m, 500 m, and 890 m depths. Only taxa with average relative abundance >1% are shown; remaining clusters (e.g., *Prochlorococcus* LLII, LLIV, HLIII, and *Synechococcus* 5.3) are grouped as "Others." Absolute abundances were estimated using spike-in quantitative metagenomics based on *recA* genes against the CyCOG v6.0 database (Diamond Blastx, e-value 1e-5, id 90%, query-cover 60%, https://zenodo.org/records/15446351).

References

1. Patel A, Noble RT, Steele JA, Schwalbach MS, Hewson I, Fuhrman JA. Virus and prokaryote enumeration from planktonic aquatic environments by epifluorescence microscopy with SYBR Green I. *Nat Protoc* 2007;**2**:269–76.
2. Noble RT, Fuhrman JA. Use of SYBR Green I for rapid epifluorescence counts of marine viruses and bacteria. *Aquat Microb Ecol* 1998;**14**:113–8.
3. Bei Q, Williams NL, Furtado LE, Blasi DD, Williams J, Brotas V, *et al.* Quantitative metagenomics for marine prokaryotes and photosynthetic eukaryotes. *ISME Commun* 2025;**5**:ycaf131.
4. Bolger AM, Lohse M, Usadel B. Trimmomatic: A flexible trimmer for illumina sequence data. *Bioinformatics* 2014;**30**:2114-20.
5. Zhang J, Kobert K, Flouri T, Stamatakis A. Pear: A fast and accurate illumina paired-end read merger. *Bioinformatics* 2014;**30**:614-20.
6. Buchfink B, Xie C, Huson DH. Fast and sensitive protein alignment using diamond. *Nat Methods* 2015;**12**:59-60.
7. Huson DH, Beier S, Flade I, Górska A, El-Hadidi M, Mitra S, *et al.* Megan community edition-interactive exploration and analysis of large-scale microbiome sequencing data. *PLoS Comput Biol* 2016;**12**:e1004957.
8. Parks DH, Chuvochina M, Rinke C, Mussig AJ, Chaumeil PA, Hugenholtz P. GTDB: An ongoing census of bacterial and archaeal diversity through a phylogenetically consistent, rank normalized and complete genome-based taxonomy. *Nucleic Acids Res* 2022;**50**:D785-94.
